# Improved Localization for 2-Hydroxyglutarate Detection at 3 T Using Long-TE Semi-LASER

**DOI:** 10.18383/j.tom.2016.00139

**Published:** 2016-06

**Authors:** Adam Berrington, Natalie L. Voets, Puneet Plaha, Sarah J. Larkin, James Mccullagh, Richard Stacey, Muhammed Yildirim, Christopher J. Schofield, Peter Jezzard, Tom Cadoux-Hudson, Olaf Ansorge, Uzay E. Emir

**Affiliations:** 1 Nuffield Department of Clinical Neurosciences, FMRIB Centre, John Radcliffe Hospital, University of Oxford, Oxford;; 2 Department of Neurosurgery, John Radcliffe Hospital, Oxford University Hospitals NHS Trust, Oxford;; 3 Nuffield Department of Clinical Neurosciences, University of Oxford, Oxford;; 4 Department of Chemistry, University of Oxford, Oxford; and; 5 Advanced Diagnostic Imaging, Philips Healthcare, The Netherlands

**Keywords:** 2-HG, semi-LASER, 3T, MRS, glioma

## Abstract

2-hydroxyglutarate (2-HG) has emerged as a biomarker of tumor cell isocitrate dehydrogenase mutations that may enable the differential diagnosis of patients with glioma. At 3 T, detection of 2-HG with magnetic resonance spectroscopy is challenging because of metabolite signal overlap and spectral pattern modulation by slice selection and chemical shift displacement. Using density matrix simulations and phantom experiments, an optimized semi-LASER scheme (echo time = 110 milliseconds) considerably improves localization of the 2-HG spin system compared with that of an existing point-resolved spectroscopy sequence. This results in a visible 2-HG peak in the in vivo spectra at 1.9 ppm in the majority of isocitrate dehydrogenase-mutated tumors. Detected concentrations of 2-HG were similar using both sequences, although the use of semi-LASER generated narrower confidence intervals. Signal overlap with glutamate and glutamine, as measured by pairwise fitting correlation, was reduced. Lactate was readily detectable across patients with glioma using the method presented here (mean Cramér–Rao lower bound: 10% ± 2%). Together with more robust 2-HG detection, long-echo time semi-LASER offers the potential to investigate tumor metabolism and stratify patients in vivo at 3 T.

## Introduction

### The Promise of 2-HG

Personalized medication in the treatment of cancer requires the discovery of reliable biomarkers and the development of tools capable of differential diagnosis according to genetic subtypes. In brain tumors, accumulation of the “oncometabolite” D-2-hydroxyglutarate (2-HG) in the majority (∼80%) of grades 2–3 gliomas and secondary glioblastomas (1, 2) is potentially such a biomarker. 2-HG is produced as a metabolic product of somatic mutations in genes encoding for isocitrate dehydrogenase (IDH) (2), particularly *IDH1* and *IDH2*, which encode for enzymes located in the cytosol and mitochondria, respectively. Recent studies have shown that patients with glioma with the IDH mutation have improved prognoses compared with those without (3, 4), and stratification by IDH mutation status and its biochemical consequences may offer new opportunities for the diagnosis, monitoring, and treatment of patients with glioma (5, 6). Therefore, accurate, noninvasive, and robust determination of 2-HG in tumors, as a surrogate marker for IDH mutation status is key for presurgical diagnosis and subsequent monitoring of treatment efficacy and relapse.

### 2-HG as a Detectable Molecule

**Figure 1.**
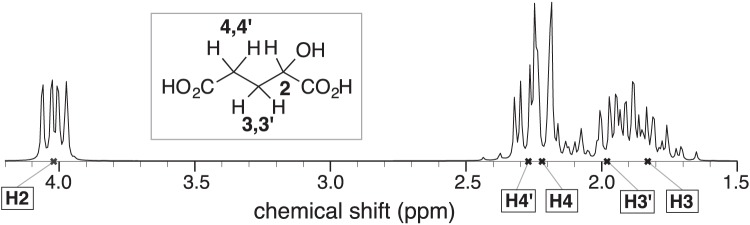
Proton spectral pattern of 2-hydroxyglutarate (2-HG) and corresponding hydrogen spins. Shown here are 3 T (127.7 MHz) with 1 Hz line broadening, 8912 spectral points, and 6 kHz spectral width. Only contributions from nonexchangeable, J-coupled spins at positions 2, 3, and 4 are visible (8).

2-HG is detectable in brain tumors using magnetic resonance spectroscopy (MRS), initially using ex vivo high-resolution magic-angle spinning (7), thereby enabling the possibility of IDH identification without the need for invasive surgical biopsy. However, the detection of 2-HG using in vivo MRS brings with it several technical challenges. 2-HG has a strongly coupled AMNPQ spin system with 5 proton resonances (H2, H3, H3′, H4, and H4′) at chemical shifts of 4.02, 1.83, 1.98, 2.22, and 2.27 ppm at pH 7 (8) (Figure 1). Pope et al., in their initial work, used a short-echo time (TE = 30 milliseconds) proton MRS point-resolved spectroscopy (PRESS) (9, 10) sequence at 3 T to demonstrate 2-HG detection in vivo (11). However, spectral overlap between the 2-HG peak at 2.25 ppm (H4 and H4′) and glutamate (Glu) and glutamine (Gln) resonances, in addition to the large baseline offset at short TE, led to a large false-positive detection rate for 2-HG. Interestingly, even at 9.4 T (where spectral resolution is ∼3 times greater), accurate short-TE detection of 2-HG in mouse models remains challenging (12), although recent success has been reported in patient studies (13). To overcome this spectral overlap issue, in vivo 2-dimensional (2D) correlation spectroscopy and spectral editing with MEGA-LASER were proposed by Andronesi et al. (14). However, such techniques can suffer from longer scan times, which are challenging to implement in clinical studies. J-editing with MEGA-LASER on the H2 and H3/H3′ scalar-coupled spins results in a difference spectrum with clear 2-HG peak at 4.02 ppm; however, in addition to the dependence of such sequences on editing efficiency, localized shimming as used in single-voxel MRS of tumors can introduce spurious water echoes around this chemical shift value (15), which could negatively influence difference spectra.

An alternative to these approaches is to use single-shot techniques with longer TE. Choi et al. optimized the timings of a PRESS sequence (TE = 97 milliseconds) to maximize the H4/H4′ peak of 2-HG at 2.25 ppm among the Glu and Gln overlaps and differentiated between IDH-mutant and wild-type (WT) tumors with much higher accuracy than with short-TE PRESS (16, 17). It is well understood that the spectra of J-coupled metabolites are susceptible to signal modulation using PRESS localization, with imperfect slice selection profiles and chemical shift displacement artifact arising from the use of narrow-bandwidth refocusing pulses (18, 19). For 2-HG, this manifests as signal loss at the H3/H3′ resonance (1.9 ppm) when using the 97 millisecond PRESS sequence (16).

### 2-HG Detection with Reduced Chemical Shift Displacement

Detection of 2-HG at 3 T may benefit from the presence of this peak at 1.9 ppm. However, this chemical shift position also lies near Glu and Gln resonances, as well as near a γ-aminobutyric acid (GABA) resonance at 1.89 ppm, and is adjacent to the N-acetylaspartate (NAA) peak at 2.01 ppm (20). Recently, an optimized semi-LASER sequence (TE = 110 milliseconds) was proposed for 2-HG detection at both 7 T and 3 T (21, 22), which, owing to increased spectral sensitivity at 7 T, is able to separate IDH1 and IDH2 mutants on the basis of spectral feature detection. This study investigates the benefit of broadband adiabatic refocusing pulses at 3 T by reducing loss of signal from the combination of poor slice localization and chemical shift displacement for the coupled spin system of 2-HG. Thus, our optimized long-TE semi-LASER localization scheme (24) is compared with an existing PRESS sequence (TE = 97 milliseconds) to detect 2-HG at 3 T in phantom and in vivo. First, quantum mechanical density matrix simulations and phantom validations are performed using both semi-LASER and PRESS (TE = 97 milliseconds) schemes to assess the benefits of adiabatic localization on the 2-HG spectral profile. The sensitivity of the optimized method is then compared in a phantom before being tested in vivo in 11 patients with glioma with and without IDH mutation.

## Methodology

### TE Optimization of Semi-LASER

**Figure 2.**
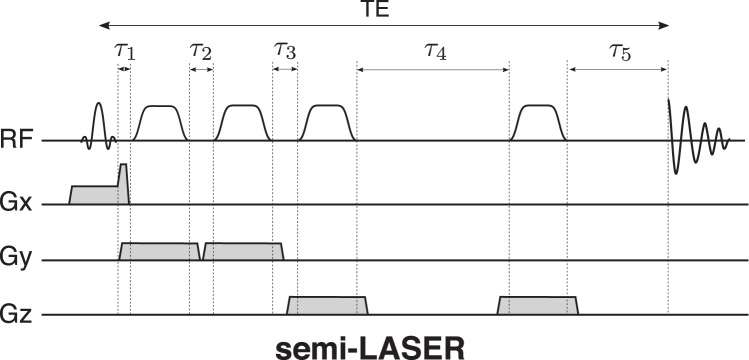
Pulse sequence diagram for semi-LASER. The total echo time (TE) is defined using interpulse delays τ; timings are given in the text.

**Figure 3.**
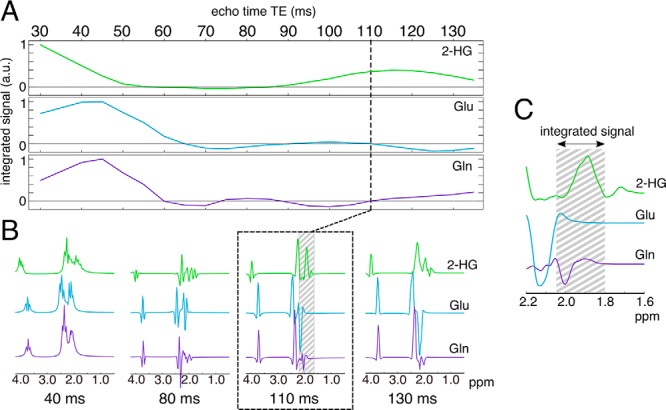
Optimization of semi-LASER TE for the detection of 2-HG. Signal integral as a function of the TE for 2-HG, glutamate (Glu) and glutamine (Gln) (A). Spectral profiles shown for 4 different TEs (B). Close up of 2-HG H3/H3′ (1.9 ppm) resonance at 110 milliseconds with overlaps showing 15 Hz integration window (C). At 110 milliseconds, semi-LASER generates near zero overlap from Glu and Gln around 1.9 ppm.

A semi-LASER localization scheme is optimized for the detection of the H3/H3′ multiplet of 2-HG among the overlapping Gln and Glu resonances. The implementation of semi-LASER is symmetrical in the positioning of its 2 pairs of gradient-selective refocusing pulses (Figure 2) (23). The TE is defined by the duration of the 90° and 180° refocusing pulses; *t*_90_ and *t*_180_; and the interpulse durations, τ, such that, τ_1_ = 0.78, τ_2_ = 1.56, τ_3_ = 1.56, τ_4_ = (TE−4*t*_180_−2τ_3_)/2 and τ_5_ = TE−τ_1_−τ_2_−τ_3_−τ_4_−4*t*_180_−*t*_90_/2 in milliseconds. Therefore, the manipulation of the TE is equivalent to varying the onset time of the final refocusing pulse (τ_4_) and readout (τ_5_). The excitation pulse in this semi-LASER implementation is an hsinc 90° excitation (duration = 2.56 millisecond, bandwidth = 3.4 kHz), and refocusing is performed using adiabatic pulses (HS4 R25, duration = 4.5 milliseconds, and bandwidth = 5.5 kHz) (24). Frequency-selective localization is simulated for the overlapping spin systems of 2-HG, Glu, and Gln on the basis of previously reported chemical shifts and coupling constants (8, 20) using real refocusing pulse information and the GAMMA/PyGAMMA simulation library of Versatile Simulation, Pulses and Analysis (VeSPA) (25). The integrated signal intensity over a 15 Hz window centered on 1.91 ppm is shown for these 3 metabolites at increasing TE (Figure 3A). It can be seen that at TE = 110 milliseconds, the H3/H3′ (1.9 ppm) peak has an upright lineshape, while overlapping signal contributions from both Glu and Gln are almost 0 (Figure 3, B and C). Signal yield of 2-HG is higher at short TE (42% at 40 milliseconds), although multiplet lineshapes and the similarity of the spectral patterns of Glu and Gln do not allow a clear distinction from 2-HG. Therefore, the TE of semi-LASER was selected as 110 milliseconds to elucidate 2-HG from these overlaps.

### Chemical Shift Simulations

For the analysis of the chemical shift displacement artifact and the effect of slice selection profile across refocusing directions on the detection of 2-HG, custom 2D quantum mechanical density matrix simulations were developed and written in Matlab (Mathworks, Inc., Natick, Massachusetts) (Pseudocode in Supplementary Materials [1]).

Several methods exist to simulate localized spectra, including simplified hard-pulse 4-compartment models, which solely account for different chemical shifts and J-modulated signals within the voxel (26), or tip-angle pulse profile models, which additionally characterize the effect of the imperfect transition bands of refocusing pulses (27). Importantly, the effect of the chemical shift and the J-evolution during a pulse is non-negligible when 1/J is comparable to the pulse duration (28). The strongest J-coupling in 2-HG, between the H4 and H4′ spins (15.0 Hz), is 1/J_44_′ = 67 milliseconds. For 2 PRESS Refoman6 refocusing pulses (duration = 13.2 milliseconds), this corresponds to nearly 40% of the J-evolution time. Thus, our localized simulations account for coupling and chemical shift evolution during the refocusing pulses.

Density matrix product operators were calculated at each stage of the pulse sequence corresponding to Hamiltonians for J-coupling, radio frequency (RF) pulse tip, and chemical shift evolution. The previously shown asymmetrical PRESS sequences (16) (TE = 97 milliseconds; TE_1_ = 32 milliseconds; and TE_2_ = 65 milliseconds; Supplementary Materials [2]) with Refoman6 refocusing pulses (duration = 13.2 milliseconds; bandwidth = 1.24 kHz; maximum B_1_ = 13.4 μT; number of points = 200) are compared with the optimized semi-LASER sequences (TE = 110 milliseconds) with full passage adiabatic refocusing pulses (HS4 R25 maximum B_1_ = 28 μT; number of points = 511). The hsinc 90° pulse was selected for excitation in both sequences. In ideal simulations, the RF pulses are modeled as instantaneous hard pulses. For the fully localized simulations, the 90° excitation pulses remain hard with 0 duration; however, the 180° pulses are divided into a series of small hard-pulse operators interspersed with the J-evolution and spatially dependent chemical shift operators to simulate slice selection gradients. Crusher gradients are simulated as incremental π/4 rotations about the z-axis positioned around each refocusing pulse similar to previous work (29, 30). To match in vivo acquisition, the RF carrier frequency is set to 2.7 ppm, and all simulations are line-broadened to 6 Hz by multiplication with an exponential factor. The sampling frequency is set at 6 kHz, and a total of 8192 time points are sampled from the resulting free induction decay (FID).

2-HG was simulated, along with 20 other commonly observed metabolites. Spin systems were simulated on a 100 × 100 grid, with a resolution of 1% of the nominal voxel size. For larger spin systems (N ≥ 6), the grid resolution was reduced to 20 × 20 resolution or 5% of the nominal voxel size to reduce computational time. Simulations were run on a computing cluster with a minimum 2.67 GHz processer and 64 GB RAM.

### Sensitivity Phantom

To test the sensitivity of our method, a test phantom containing 6 bottles of varying 2-HG concentrations (66 ml) (or R,S-α-hydroxyglutaric acid, Sigma Aldrich, St. Louis, MO) was constructed. 10mM of glycine (Gly) was included in each bottle to act as a reference peak, having only a singlet resonance at 3.55 ppm (20). Concentrations of 2-HG were 0.1, 0.5, 1, 2, 5, and 8mM. Each bottle was buffered with phosphate-buffered saline, and average pH was measured to be 7.5 ± 0.1. The larger bottle was filled with NaCl solution. Phantom measurements were acquired on a 3 T whole-body magnetic resonance (MR) Prisma system (Siemens, Erlangen, Germany) with a 32-channel receive array head coil. Voxels of 20 × 20 × 20 mm^3^ volume were obtained and shimmed to second order using GRESHIM (31). Both PRESS (TE = 97 milliseconds) and optimized semi-LASER (TE = 110 milliseconds) spectra were acquired with VAPOR water suppression and outer volume saturation bands (32) at a TR of 3 seconds. RF pulses and timings were equivalent to those in simulations, and 128 transients were acquired for each bottle in the phantom together with an unsuppressed water reference used for eddy current correction.

### In Vivo MRS

In total, 11 patients with glioma (male, 7; female, 4; age, 39 ± 11 years) underwent scanning with the optimized semi-LASER (TE = 110 milliseconds) sequence in addition to PRESS (TE = 97 milliseconds). Of those, 6 patients underwent scanning with semi-LASER only because of constraints during a time-sensitive MR protocol. Written and informed consent approved by the South Central-Oxford B National Research Ethics Committee was obtained from all participants. Scanning was performed before resection and histological confirmation in all but 1 patient (P08), who underwent only partial resection and whose residual tumor was scanned early postoperatively. To test both methods, 5 healthy volunteers (male, 2; female, 3; age, 28 ± 4 years) also underwent scanning with both sequences. Voxels of 20 × 20 × 20 mm^3^ volume were acquired on 3 T whole-body MR Prisma/Trio systems, and the protocol remained identical to the phantom investigation with 64–96 transients collected in patients and 96 in healthy volunteers. An unsuppressed water scan was also collected for absolute quantification.

### Immunohistochemistry and DNA Sequencing

Immunohistochemistry was performed on (4 μm) tissue sections to detect the R132H IDH mutation. Endogenous peroxidase activity was blocked and epitopes retrieved (autoclave, 121°C for 10 minutes in 10mM sodium citrate; pH 6.0). Visualization was achieved using Dako REAL EnVision/HRP, Rabbit/Mouse (ENV) kit (Dako), after incubation of sections with the primary antibody (IDH1 R132H, clone H09, Dianova, 1:100, overnight at 4°C). DNA sequencing was performed on regions of IDH1- and IDH2-containing codons 132 and 172, respectively (amplified with Polymerase chain reaction (PCR)). Products were examined using agarose gel separation and then purified (MinElute PCR Purification Kit, Qiagen). Sequencing reactions were achieved using BigDye Terminator chemistry and an ABI-3730 sequencer. The methodology is identical to that reported by Emir et al. (2016) (21).

### Analysis

Basis sets are generated from the fully localized simulations for metabolite assignment with LCModel (33). The basis set includes 2HG, alanine (Ala), ascorbate (Asc), Asp, GABA, Cr+PCr (total creatine: tCr), Gln, Glu, Gly, myo-inositol (myo-Ins), lactate (Lac), NAA+NAAG (total NAA: tNAA), phosphocholine (PCho), phosphatidylethanolamine (PE), scyllo-inositol (scyllo-Ins), taurine (Tau), glucose (Glc), and glutathione (GSH). Individual spectra were corrected for frequency and eddy current. For quantification of absolute metabolite concentrations, an exponential scaling correction, based on the transverse decay rate, T2, was applied to the unsuppressed water reference (assuming a bulk water concentration of 55.5mM) using the average of recently published values in low- and high-grade tumors (150 milliseconds) (34) and was set to 83 milliseconds for healthy brain (35). A correction based on metabolite relaxation rates (T1 and T2) was not performed in this work. Thus, all reported absolute concentrations are semiquantitative in nature.

To assess the effect of overlaps on spectral fitting with the LCModel, correlation coefficients are reported as the average of the pairwise correlation between individual metabolite fits *X* and *Y*, *r*_*X:Y*_. Signal-to-noise ratio (SNR) is reported as the LCModel output ratio of maximum signal height to 2× the root mean square of the fitting residual. In this work, 95% confidence intervals (≈2× Cramér–Rao lower bound [CRLB]) are reported alongside the weighted mean of the absolute concentration values as described in the LCModel manual (33).

## Results

### PRESS and Semi-LASER Localization

**Figure 4.**
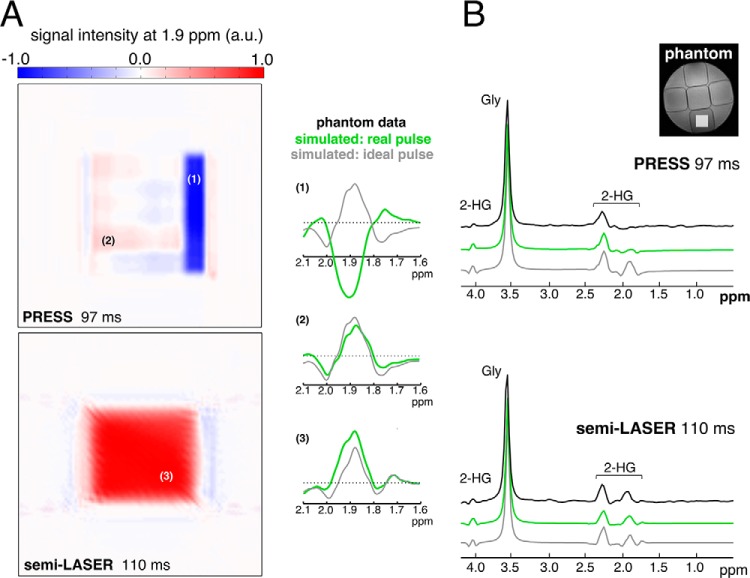
2-HG localization using point-resolved spectroscopy (PRESS) and semi-LASER. 2-dimensional (2D) grid simulation showing integrated H3/H3′ peak intensity at 1.9 ppm (15 Hz window) for both sequences (A). 200 × 200 spatial grid size. Spectra taken from representative positions (1–3) within grids are shown alongside and show deviations from ideal pulse simulations. Total simulated 2-HG acquisition (ideal and localized) shown alongside data taken from phantom (black); Gly is also included (B). The H3/H3′ resonance appears to be diminished using real refocusing pulses in PRESS because of large compartmental chemical shift displacement artifacts; however, it remains upright and close to the ideal with semi-LASER.

First, localization of the 2-HG H3/H3′ multiplet was investigated using simulations of our optimized semi-LASER sequence (TE = 110 milliseconds) and the original PRESS (TE = 97 milliseconds) sequence (16) (Figure 4). The total integrated H3/H3′ peak 2-HG intensity at 1.9 ppm, following 2D fully localized simulations (green), is shown at each position on the 200 × 200 simulated grid on the intensity maps in Figure 4A. A heterogeneous distribution of signal intensity at 1.9 ppm, which manifests as compartments of several distinct signal profiles, is seen using PRESS localization. In particular, there is a complete inversion of the 1.9 ppm peak within one of these compartments (Figure 4A (1)), accounting for ∼19% of the total 2D voxel, which has acquired an antiphase signal characteristic compared with the ideal pulse simulation (gray). Only ∼52% of the localized PRESS voxels contain a positive signal contribution at this shift value (Figure 4A (2)). However, a semi-LASER sequence displays a far superior slice selection profile and reduced chemical shift displacement artifact for the multiplet of H3/H3′. This is evident from the presence of a single, uniform compartment containing positive signal contribution, and it is almost identical to the ideal pulse simulations (Figure 4A (3)).

Figure 4B shows the spectral comparison between 2-HG and Gly simulated using ideal (gray) and fully localized simulations (green) and the spectrum acquired in the phantom test bottle containing 2mM 2-HG and 10mM Gly (black). Using PRESS localization, there is a loss of the H3/H3′ resonance as shown in both the localized simulations and phantom data, which is in strong contrast to ideal simulations. This peak is preserved using semi-LASER at TE of 110 milliseconds, which generates near-identical spectral profiles across ideal, localized, and phantom data.

Comparing the fully localized 2-HG spectra with a simulated pulse-acquire experiment, the signal amplitude at 2.25 ppm is measured as 53.5% using PRESS and 37.9% using semi-LASER (neglecting T2 relaxation). At the 1.9 ppm resonance (optimized for in this study), this becomes 2.9% for PRESS and 36.4% for semi-LASER.

### Sensitivity of 2-HG Detection

**Figure 5.**
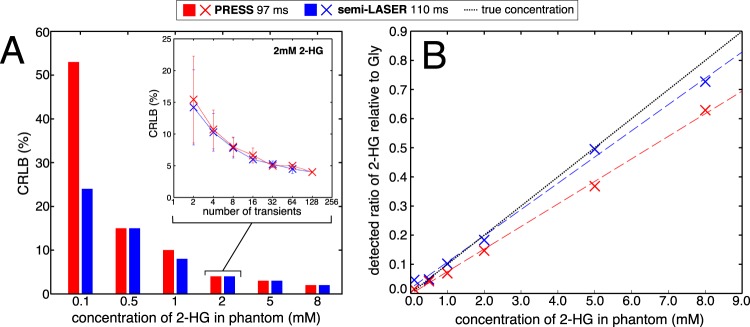
Sensitivity of semi-LASER and PRESS for 2-HG detection in phantom. Cramér–Rao lower bound (CRLB) of the LCModel fitting as a function of 2-HG concentration collected for 128 transients (A). Both methods show comparable errors across all concentration values. In addition, CRLBs are shown as a function of the number of acquired averages in a phantom bottle containing 2mM 2-HG. The ratio of detected 2-HG to Gly is plotted as a function of concentration compared with true concentration (B). Absolute values are not reported because of T2 decay time differences in phantom.

The *sensitivity* of both methods to detect 2-HG across a range of concentrations was assessed next in the phantom. Spectra were line-broadened (6 Hz) to match in vivo conditions. Figure 5A shows the CRLB of fitting the acquired spectra with the simulated localized LCModel basis. Both PRESS and semi-LASER maintain near-identical CRLBs of 2-HG fitting for concentrations >0.5mM, falling to 2% for 8mM of 2-HG (SNR: 57 semi-LASER; 64 PRESS). At 0.1mM, the CRLB after 128 averages is 53% and 19% for PRESS and semi-LASER, respectively. To further examine the sensitivity of the methods, data were split into averaged spectra containing 2, 4, 8, 16, 32, 64, and 128 transients. Figure 5A also shows the LCModel CRLB output as a function of the acquired number of averages, using the 2mM 2-HG phantom bottle as an example. Both methods maintain identical relationships with an increasing number of spectral averages. Even with only 2 averages (SNR ∼ 15), both methods fit 2-HG at 2mM with CRLBs of 15% ± 7% and 14% ± 6% for PRESS and semi-LASER, respectively.

Figure 5B shows the ratio of detected 2-HG to Gly with the LCModel over the same concentration values without correcting for T2 differences. For concentrations >0.1mM (CRLBs <20%), both methods differed significantly in their estimation of 2-HG concentration(*P* < .008, paired *t* test) with average detection ratios of 76% ± 6% and 96% ± 6% of the true ratio for PRESS and semi-LASER, respectively.

### Acquisition In Vivo

**Figure 6.**
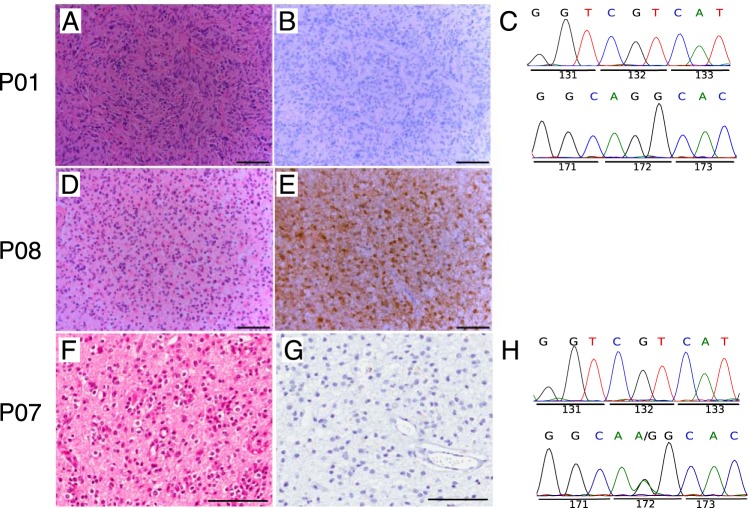
Tumor immunohistochemistry and sequencing. Hematoxylin and eosin (A, D, F); immunohistochemistry using anti-IDH1 R132H antibody (brown reaction product) (B, E, G); and PCR and direct sequencing of codon 132 of IDH1 (top trace) and R172 of IDH2 (bottom trace) (C, H). Case P01 (A, B, C): glioblastoma without IDH1 R132H mutation or IDH2 mutation; case P08 (D, E): astrocytoma with IDH1 R132H mutation; case P07 (F, G, H): oligodendroglioma without IDH1 R132H mutation but with IDH2 R172K mutation (P07; histological data previously published in the study by Emir et al.) (21): all scale bars are 100 μm.

**Figure 7.**
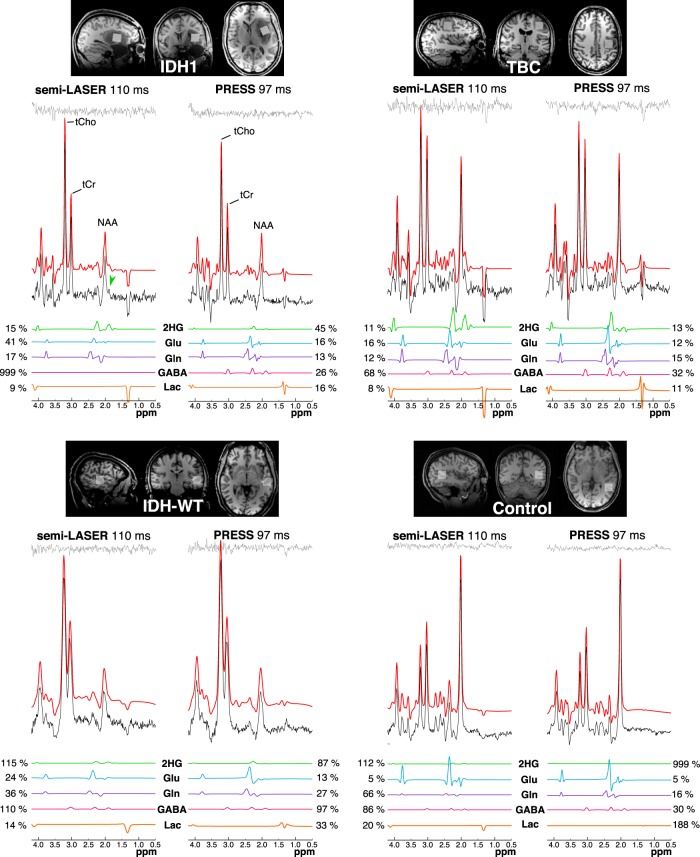
Example of in vivo spectral acquisition using semi-LASER and PRESS. Data shown are from IDH-mutant (IDH1), To be confirmed (TBC), IDH wild-type (IDH-WT), and healthy volunteer (control). The LCModel fits of 2-HG, Glu, Gln, GABA, and lactate (Lac) shown with CRLBs (%) are given next to corresponding spectra. The green arrow in IDH1 indicates 2-HG peak visible with semi-LASER. Major peaks of NAA, total choline (tCho), and total creatine (tCr) are labeled on the IDH1 spectra for reference.

Data were then acquired in vivo and analyzed from our sample of 11 patients with glioma and 5 healthy controls using semi-LASER (TE = 110 milliseconds, n = 11 patients) and PRESS (TE = 97 milliseconds, n = 7 patients). The summary of these data for 2-HG detection is given in Table 1 (information on further metabolites is given in Supplementary Materials [3]). Figure 6 shows the immunohistochemistry and DNA-sequencing results from 3 patients. Further, 4 examples of the in vivo spectral quality along with the LCModel fits are shown in Figure 7—one example for each of the following: IDH1-mutant patient (P08); IDH-WT (P03), to be confirmed (TBC; P10), and healthy volunteer (control; C01). These spectra are representative of the high spectral quality obtained with both sequences. In particular, the amplitude of the residual water peak is less than tNAA or tCr, and no significant lipid contamination was observed, owing to the use of outer volume suppression. This resulted in flat baselines over the analysis range of 0.5-4.2 ppm except in 1 patient (P06), for whom spectral analysis was restricted to 3.8 ppm.

**Table 1.** Summary of *in vivo* Data for 11 Glioma Patients and 5 Healthy Controls with 2-HG Concentrations (mM) and CRLBs Including PRESS were Obtained

ID	Sex/Age	Tumour Type/WHO Grade	IDH-Mutant	2HG (mM)			
				Semi-LASER		PRESS	
				Conc.	CRLB %	Conc.	CRLB %
P01	M/53	Glioblastoma/IV	IDH-WT	1.25	40	NA	
P02	F/37	Glioblastoma/IV	IDH-WT	0.88	34	0.00	999
P03	M/45	Oligodendroglioma/III	IDH-WT	1.17	115	1.55	87
P04	M/52	Astroglial Tumour/III	IDH1	3.87	15	NA	
P05	M/33	Anaplastic Astrocytoma/III	IDH1	0.59	68	NA	
P06	M/22	Diffuse Astrocytoma/II	IDH1	1.95	21	2.74	22
P07	F/26	Oligodendroglioma/II	IDH2	4.46	8	NA	
P08	F/56	Astrocytoma/III	IDH1^a^	1.78	15	0.78	45
P09	F/44	TBC	TBC	1.94	16	1.63	69
P10	M/24	TBC	TBC	2.91	11	3.81	13
P11	M/36	TBC	TBC	6.33	8	6.06	8
C01	F/30	—	—	0.32	112	0	999
C02	M/35	—	—	0.43	103	0	999
C03	F/24	—	—	0.66	59	0	999
C04	F/25	—	—	0	999	0	999
C05	M/24	—	—	0.14	228	0	999

IDH1 or IDH2 indicates mutations in either enzyme.

^a^ denotes post-operative scan.

Abbreviations: TBC, To be confirmed; NA, Not Applicable (Data not acquired).

The presence of the H3/H3′ multiplet can be clearly seen in the raw IDH1 tumor spectrum (Figure 7) and in a further 6 patients (including the TBC patient, also shown in Figure 7) who underwent scanning with semi-LASER (Supplementary Materials [4]). The occurrence of this peak at 1.9 ppm, upfield of the NAA singlet at 2.01 ppm, gives in vivo 2-HG semi-LASER spectra a distinctive spectral profile and can be seen in the raw data even before the LCModel fitting.

In the 5 histologically confirmed IDH-mutated tumors, semi-LASER CRLBs of 2-HG fitting of ≤21% were obtained in all but 1 patient (P05: 68%). For PRESS, only 1 data set has been histologically confirmed as IDH-mutated. Because of this low number, a comparison using conventional CRLB rejection thresholds would introduce bias. Therefore, the average 95% confidence interval for 2-HG in IDH-mutants was compared in patients who underwent scanning using both methods (n = 2) and concentrations of 1.3 ± 1.0mM and 1.8 ± 0.7mM for PRESS and semi-LASER, respectively, were found. If we include patients with IDH status yet to be confirmed in this analysis (n = 5), these confidence intervals become 2.8 ± 1.2mM and 2.5 ± 0.7mM for PRESS and semi-LASER, respectively. In both cases, detected concentrations are in agreement across methods; yet, the confidence intervals are considerably improved using the optimized semi-LASER. For the IDH-WT patients, the minimum fitted CRLB is 34% for semi-LASER and 87% for PRESS. In all healthy controls, 2-HG remained correctly undetected (CRLBs, 999%) using PRESS and also with semi-LASER all CRLBs were >100%, except 1 (minimum, 59%; maximum, 999%).

Thus, a patient (P10) (Figure 7) awaiting biopsy is predicted to be IDH-mutated given the low CRLB (11%) using semi-LASER and the clear peak in the spectrum at 1.9 ppm. Glu and Gln are detectable with both methods; however, Glu is detected with higher CRLBs using semi-LASER than PRESS. In addition, at 110 milliseconds, the Lac peak at 1.33 ppm is almost fully inverted for semi-LASER, which significantly aids in its fitting, generating much lower average CLRBs across all patients of 10 ± 2% compared with 21 ± 8% for PRESS.

**Figure 8.**
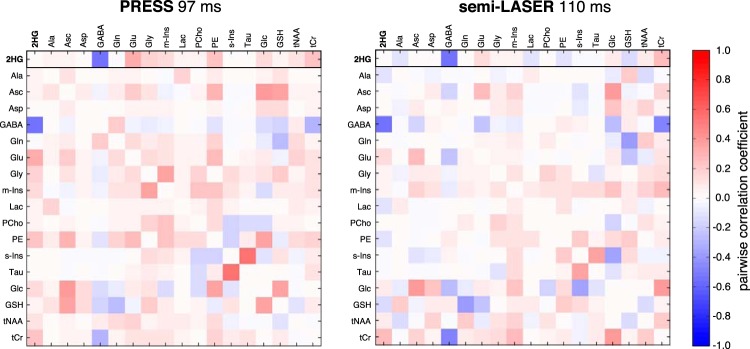
Average pairwise correlation matrices of the LCModel metabolite fitting across patients with glioma showing PRESS (n = 7 patients) and semi-LASER (n = 11 patients). 2-HG is highlighted as the first entry in the matrix.

Figure 8 shows the average correlation coefficients across all metabolite fittings and patients for each sequence. It can clearly be seen that using both sequences, the pairwise correlation coefficients between 2-HG and GABA are large (semi-LASER: *r*_*2HG:GABA*_ = −0.55 ± 0.26; PRESS: *r*_*2HG:GABA*_ = −0.53 ± 0.14). Similar measures of the correlation coefficients for Glu are as follows: semi-LASER: *r*_*2HG:Glu*_ = 0.14 ± 0.16; PRESS: *r*_*2HG:Glu*_ = 0.31 ± 0.19), and for Gln, the coefficients are as follows: semi-LASER: *r*_*2HG:Gln*_ = 0.01 ± 0.09; PRESS: *r*_*2HG:Gln*_ = −0.02 ± 0.06.

## Discussion

The results reveal that an optimized semi-LASER MRS sequence offers high sensitivity and localization for in vivo detection of 2-HG, characteristic of IDH-mutated brain tumors, at 3 T. The use of a long TE (110 milliseconds) not only enables direct detection of the H3/H3′ multiplet of 2-HG at 1.9 ppm in the in vivo spectra but also generates reduced fitting correlations with overlapping Glu and Gln resonances and improved fitting of Lac compared with an existing long-TE PRESS sequence (16). These improvements promise a more robust assignment of such metabolites for the investigation of tumor metabolism in vivo.

The need to consider the effect of localization on the spectral pattern of the strongly coupled spin system of 2-HG is strikingly shown using 2D simulations (Figure 4). It has been shown that the loss of the H3/H3′ multiplet with PRESS, as eluded to by Choi et al. (17), is because of a significantly large compartmental artifact within the voxel, which arises from long J-evolution times under enhanced chemical shift displacement from narrow-bandwidth RF pulses. This effect manifests as the formation of a large negatively phased signal component in the PRESS voxel, opposite to that expected using ideal pulses, which has a deleterious effect on any positive signal contribution from other compartments. The effect of signal cancellation using PRESS has been well documented for several spin systems (18, 28), and it is known that for strongly coupled metabolites in particular, coherence transfer and spin evolution become harder to predict (19). Comparatively, semi-LASER suffers from considerably reduced compartmental artifacts, with an overall 2-HG spectral pattern that is similar to that expected using ideal hard pulses (Figure 4). This finding is similar to that reported by Kaiser et al. (30), namely that using broadband adiabatic localization on strongly coupled spins retains much of the ideal spectral pattern. In this study, the chemical shift displacement across a single dimension (measured between the H3/H3′ peak and the carrier frequency at 2.7 ppm) is 8.2% of the nominal voxel size using the Refoman6 pulses in PRESS and 1.9% using the 5.5 kHz HS4 R25 adiabatic pulse in semi-LASER. The chemical shift displacement can be improved even further, by refocusing using frequency offset corrected inversion (FOCI) or gradient offset independent adiabatic (GOIA) (36, 37) pulses, with the potential benefit of reducing total specific absorption rate (SAR) (38). Even when the B_1_ requirement of semi-LASER is reduced to match that of PRESS, using longer-duration pulses (10.2 milliseconds, maximum B_1_ = 13.4 μT), the signal amplitude at 1.9 ppm is still ∼12 times larger because of the increased bandwidth (2.46 kHz) of the broadband refocusing pulses. It is worth noting from a practical viewpoint that a strong chemical shift displacement artifact not only results in signal attenuation, as discussed above, but also causes metabolite measurement displacement from the prescribed voxel location, which is potentially problematic in the spatially heterogeneous environment of a tumor.

Our optimization on the H3/H3′ 2-HG, Gln, and Glu overlaps resulted in a TE which is 13 milliseconds longer than that for PRESS. Considering J-modulation alone, this resulted in H4/H4′ (2.25 ppm) 2-HG peak amplitudes to be 29% smaller using semi-LASER than using PRESS. This discrepancy did not appear to affect semi-LASER detection in the phantom. Indeed, CRLBs were similar for both sequences as a function of 2HG concentration and the number of acquired averages. When comparing the detected 2HG-to-Gly ratios in the phantom with true values, semi-LASER appeared to detect more accurate concentrations than PRESS, although both methods tended to underestimate. This is in contrast with the results of the study by Choi et al. who correctly detected true phantom concentrations within the same range using the PRESS 97 millisecond sequence (17). In the study presented here, only relative concentration ratios in the phantom are reported, as a correction for T2 relaxation was not performed. T2 relaxation values of 2-HG and Gly in the phantom would be difficult to estimate, yet it would account for the underestimation of concentration in both methods resulting from fitting on reduced signal amplitudes. The observation that concentrations were underestimated more using a shorter-TE PRESS sequence than semi-LASER could be explained by the fact that during localization with 4 refocusing pulses in semi-LASER, apparent signal decay from T2 relaxation is somewhat reduced (39). Micheali et al. have measured apparent T2 values for NAA and Cr at 4 T using CP-LASER and CP-PRESS and found values almost twice as large (40).

Although the H4/H4′ (2.25 ppm) resonance is smaller in amplitude with semi-LASER, the presence of the H3/H3′ resonance at 1.9 ppm helps with detection. This is particularly true at very small concentrations of 0.1mM, where the CRLB was 19% for semi-LASER and 53% for PRESS. Although not realistically detectable in vivo, detection at these low concentrations in the phantom indicate that semi-LASER may offer improvements on detectability at small concentrations of 2-HG. It is hoped that this increase in sensitivity would benefit measurement from small tumor volumes, which have proven problematic for 2-HG detection in a recent study using PRESS (TE = 97 milliseconds) (41).

In the in vivo acquisitions, the presence of the H3/H3′ peak is clearly apparent alongside the NAA singlet in the IDH1-mutated patient shown in Figure 7 (green arrow) and also in 6 other patients (3 IDH-mutant, 3 TBC; Supplementary Materials [4]). The identification of this peak in the raw spectra presents a clear benefit to rapidly ascertain IDH status by obviating the need for more complex fitting algorithms. The proximity of H3/H3′ to NAA at 2.01 ppm is 14.1 Hz at 3 T; thus, if metabolite line widths were around this order of magnitude or NAA concentrations were high (for example, in lower-grade gliomas) (42), this peak would become visually unresolvable. However, in all our patients, reasonable line widths were obtained and an inspection of the average pairwise fitting correlations between total NAA and 2-HG revealed very low average coefficients (*r*_*2HG:tNAA*_ = 0.09) (Figure 8).

The narrower confidence interval on the estimation of 2-HG concentration in IDH-mutated tumors using semi-LASER (1.8 ± 0.7 mM) compared with that using PRESS (1.3 ± 1.0 mM) is also maintained after the inclusion of patients whose IDH mutation status is yet to be confirmed (PRESS: 2.8 ± 1.2 mM; semi-LASER: 2.5 ± 0.7 mM). A direct comparison between semi-LASER and PRESS detection rates, using conventional CRLB rejection thresholds, was not appropriate in this study (low number of patients). However, the use of 95% confidence intervals, derived from CRLBs, revealed that estimated 2-HG concentrations using semi-LASER are in agreement with those using PRESS and that semi-LASER yielded more consistent concentrations as determined by the narrower confidence intervals. Therefore, this optimized semi-LASER sequence may offer an increase in the reliability of 2-HG estimation, although investigation on a larger number of IDH patients is needed.

A correlational analysis revealed that, on average, the pairwise fitting correlation is larger for Glu using PRESS (*r*_*2HG:Glu*_ = 0.31) than for that using semi-LASER (*r*_*2HG:Glu*_ = 0.14). This is attributed to the additional “degree of fitting” on the H3/H3′ resonance where Glu overlap is reduced. However, CRLBs of fitting for Glu across patients and controls were higher for semi-LASER (29%) than for PRESS (18%). The reason for this might be 2-fold. First, accurate detection of low Glu concentrations using semi-LASER could result in larger CRLBs of detection. It is important to note that larger CRLBs do not necessarily equate to inestimable metabolite concentrations, and care should be taken to reject results with larger CRLBs as an inability to detect (43). Interestingly, a recent in vitro study on the metabolic profile of IDH mutants revealed a decrease in Glu levels (44). Second, a large 2-HG concentration, which lies on top of reduced Glu and Gln resonances at 1.9 ppm, could lead to an underestimation of these lower-concentration metabolites. Further investigation in phantom may help understand the ability of the proposed method to determine Glu concentrations.

A limitation of this study is the reporting of absolute metabolite levels quantified using an internal water reference within tumor voxels. Factors which will affect this quantification method, such as regional differences in water concentration and metabolite relaxation in the heterogeneous tumor environment, are difficult to estimate in practice during patient studies. Apparent metabolite T2 decay rates are variable with both tumor grade and MRS sequence used, and have the potential to complicate the quantification of metabolite concentrations (45). There is a need for accurate measurement of T2 relaxation rates of 2-HG and other metabolites using semi-LASER at 3 T.

The low CRLBs (≤40%) of 2-HG in 2 IDH-WT tumors using semi-LASER indicate that this method may lack specificity in certain cases. The reason for these false-positive assignments may be because of the failure of the LCModel to fit GABA instead of 2-HG. Indeed, for one particular patient (P02; IDH-WT), 2-HG was fitted with CRLB of 34%, yet GABA was not fit at all. The pairwise correlation coefficient of fitting between 2-HG and GABA is large and negative (*r*_*2HG:GABA*_ = −0.55) for semi-LASER, suggesting that 2-HG and GABA are “stealing” signals from each other. This could explain the lower average CRLBs for 2-HG in healthy volunteers in whom semi-LASER was used. It is worth noting that PRESS is also not immune to this effect given its correlation of *r*_*2HG:GABA*_ = −0.53.

The unambiguous assignment of the H3/H3′ peak also relies on the fact that GABA concentration at this position is relatively small given its resonance at 1.89 ppm; 2-HG concentrations typically range from 5–35 μM in gliomas (2); therefore, 2-HG tends to dominate the spectrum. However, the presence of GABA in human gliomas is unclear. One ex vivo study of several tumor types did not report any GABA detection (46), while a separate ex vivo study found an increase in GABA in low-grade, IDH-mutated gliomas (47). In the in vivo work done by Choi et al., GABA was not detectable or existed at a very low levels across subjects (17). In a recent study, using an optimized PRESS sequence at 7 T, Ganji et al. examined the effect of overlaps by removing 2-HG from the fitting basis, which resulted in a considerable influence on the detection of GABA along with Glu and Gln (48). However, removing GABA from the basis set may lead to substandard metabolite fitting for early-stage tumors or voxels containing healthy tissue. It would be an interesting avenue for future research to use GABA-specific MRS sequences to assess concentrations of this metabolite in vivo in different tumor types.

A significant benefit of the 110 millisecond optimized TE of semi-LASER is that Lac was quantifiable across all patients and controls with significantly reduced CRLBs compared with PRESS (*P* = 0.038, paired *t* test). At this TE, the Lac doublet at 1.3 ppm is almost fully inverted (1/J = 144 millisecond; 49), which allowed accurate estimation with semi-LASER. Importantly, the acquisition of Lac along with 2-HG is an interesting prospect because of its role in tumor metabolism (50). Similar to the recent observations at 7 T (21), elevated Lac levels were detected across all patients with tumors compared with controls (2.3×, *P* = 0.003, paired *t* test). Furthermore, the single confirmed IDH2 patient in the current study had the largest detected 2-HG concentration (4.46mM) relative to others, lending support to the putative link between 2-HG levels and IDH mutation type mentioned previously (21). However, more reliable acquisition of 2-HG with lower CLRBs of fitting, as well as other tumor metabolites such as Gly and glutathione, may better be achieved at ultrahigh fields (≥7 T) (13, 21, 48, 51), where gains from spectral resolution reduce problems with overlap. In summary, the detection of multiple tumor-related metabolites, along with accurate estimations of 2-HG, could help build a more integrated picture of cellular metabolic processes in vivo and lead to further stratification of patients. For 3 T semi-LASER at 110 milliseconds, wider, cross-centre studies are required on larger patient groups to fully assess its ability to achieve this goal.

## Conclusion

An optimized semi-LASER sequence at 3 T with long TE (110 milliseconds) is capable of a robust 2-HG detection in IDH patients. Because of its reduced chemical shift displacement artifact compared with that of an existing PRESS localization scheme, compartmental artifacts are considerably reduced, leading to the complete refocusing of a resonance at 1.9 ppm. This distinct peak is clearly observable in the in vivo spectrum and together with reduced Glu and Gln overlaps, aids in the assignment of 2-HG. Lactate is also easier to detect with this method compared with PRESS because of the slightly longer TE. Because novel therapeutic approaches to glioma treatment are currently being developed, such as the targeted inhibition of IDH-mutants (52), it is anticipated that this method will be hugely beneficial for accurate in vivo assessment of tumor status and glioma progression at 3 T through robust 2-HG and Lac estimation.

### Supplemental Materials

Supplementary Materials [1]–[4]
