# Supplementary material for: Improved Localization for 2-Hydroxyglutarate Detection at 3 T Using Long-TE Semi-LASER
Source: Tomography. 2016 Jun;2(2):94–105. doi: 10.18383/j.tom.2016.00139 (PMC4990123; doi:10.18383/j.tom.2016.00139)
Supplement: Supplementary Materials [1]–[4] [file tom-00139-16-s001.pdf]

Supplementary Materials:
Improved localisation for 2-hydroxyglutarate detection at 3T using long-TE semi-LASER

[1] Density Matrix Simulation Pseudocode

Density Matrix simulations were based on the density operator approach [1], which states that evolution of the density matrix σ over time t, can be written

$$\frac{d\sigma}{dt} = -i[H(t), \sigma(t)], \tag{1}$$

for Hamiltonian H, describing chemical shift, J-coupling and time-dependent RF and has a solution

$$\sigma(t + t_1 + t_2 + \ldots + t_k) = e^{-iH_k t_k} \ldots e^{-iH_2 t_2} e^{-iH_1 t_1} \sigma(t) e^{iH_1 t_1} e^{iH_2 t_2} \ldots e^{iH_k t_k}, \tag{2}$$

for time intervals t_1, t_2 ... t_k. Evolution of the spin density matrix can therefore be described over intervals t_k and calculated using the propagators $e^{-iH_k t_k}$.

Pseudocode of localisation using 180°_x pulse and free evolution

```
N = 200                                                    ▷ Set the number of 2D grid points
σ_0 = initial density matrix
pulse = read in RF pulse information
for x = 1 : N do
    for y = 1 : N do                                       ▷ Loop over spatial grid

        ω_{x,s} = δ_s[Hz] + γGx/2π
        ω_{y,s} = δ_s[Hz] + γGy/2π                         ▷ Frequency offset of spin s at x, y with shift δ

        for c = 0 : π/2 : 3π/2 do                          ▷ Crusher Gradients
            σ_c ← e^{-iH_crush(c)} σ_0 e^{iH_crush(c)}
            for n = 1 : length(pulse) do                   ▷ Loop over points in 180° pulse
                σ_c ← e^{-iH_RF(n)t_RF} σ_c e^{iH_RF(n)t_RF}          ▷ RF evolution at point n for t_RF
                σ_c ← e^{-iH_CS(ω_{x,s})t_RF} σ_c e^{iH_CS(ω_{x,s})t_RF}  ▷ Chemical shift at x, for t_RF
                σ_c ← e^{-iH_J t_RF} σ_c e^{iH_J t_RF}              ▷ J-evolution for t_RF
            end for
            σ_c ← e^{-iH_crush(c)} σ e^{iH_crush(c)}
            σ += σ_c
        end for
        σ ← σ/4                                            ▷ Normalise after 4-fold cycle of crusher gradients

        σ ← e^{-iH_free τ} σ e^{iH_free τ}                  ▷ Free evolution for τ
    end for
end for
```

The 90° pulses are modeled as hard pulses in our simulations

[1] O. Sørensen, G. Eich, M. Levitt, G. Bodenhausen, and R. Ernst. Product operator formalism for the description of NMR pulse experiments. *Progress in Nuclear Magnetic Resonance Spectroscopy*, 16:163–192, 1984.

[2] PRESS Sequence Diagram

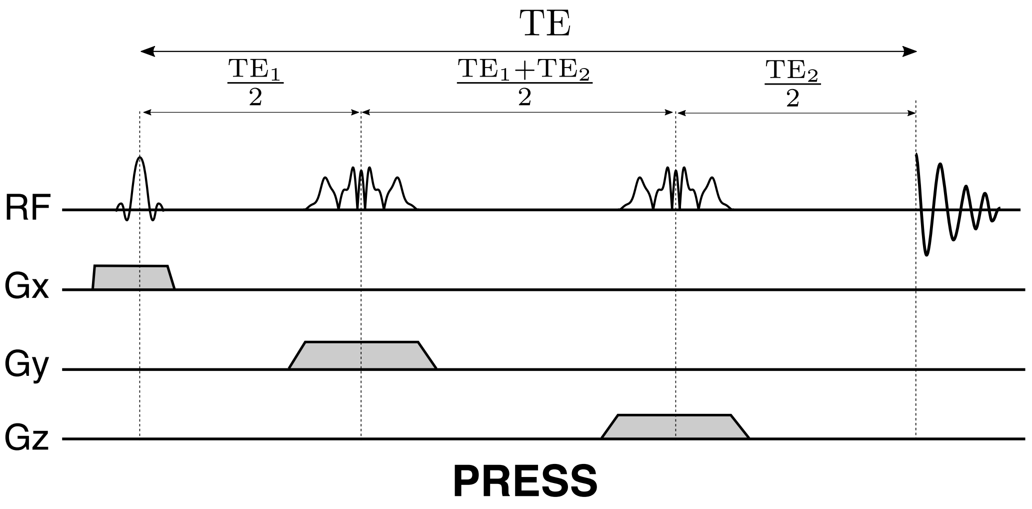

Figure 1: Asymmetric PRESS localisation scheme with characteristic sub-echo times TE_1 = 32 ms, TE_2 = 65 ms and total echo time TE = 97 ms as proposed by Choi et al. [1].

Fig. 1 shows the PRESS method used for comparison in this work, with Refoman6 refocussing pulses. Total echo time TE is 97 ms and two asymmetric sub-echoes are TE_1 = 32 ms and TE_2 = 65 ms. Crusher gradients were also applied around refocussing pulses. A standard hsinc 90° excitation pulse (duration = 2.56 ms, bandwidth = 3.4 kHz) is used in this work, although Choi et al. originally made use of an amplitude/frequency modulated excitation pulse.

[1] C. Choi, S. K. Ganji, R. J. DeBerardinis, K. J. Hatanpaa, D. Rakheja, Z. Kovacs, X.-L. Yang, T. Mashimo, J. M. Raisanen, I. Marin-Valencia, J. M. Pascual, C. J. Madden, B. E. Mickey, C. R. Malloy, R. M. Bachoo, and E. A. Maher. 2-hydroxyglutarate detection by magnetic resonance spectroscopy in IDH-mutated patients with gliomas. *Nature medicine*, 18(4):624–9, 2012.

[3] Metabolite Concentration Table

				Concentrations (mM)											
				Glu				GABA				Lac			
				semi-LASER		PRESS		semi-LASER		PRESS		semi-LASER		PRESS	
ID	Sex/Age	Tumour Type / WHO Grade	IDH-mutant	Conc	CRLB	Conc	CRLB	Conc	CRLB	Conc	CRLB	Conc	CRLB	Conc	CRLB
P01	M/53	Glioblastoma / IV	IDH-WT	2.25	16	NA		0.10	268	NA		0.84	11	NA	
P02	F/37	Glioblastoma / IV	IDH-WT	2.57	11	4.26	8	0.00	999	0.97	23	0.62	11	1.46	18
P03	M/45	Oligodendroglioma / III	IDH-WT	4.46	24	9.45	13	0.77	110	1.03	97	1.18	14	4.40	33
P04	M/52	Astroglial Tumour / III	IDH1	1.74	24	NA		0.17	188	NA		1.02	9	NA	
P05	M/33	Anaplastic Astrocytoma / III	IDH1	1.71	17	NA		0.01	999	NA		0.70	11	NA	
P06	M/22	Diffuse Astrocytoma / II	IDH1	0.93	50	2.21	23	0.00	999	0.30	137	0.63	13	1.89	29
P07	F/26	Oligodendroglioma / II	IDH2	0.43	58	NA		0.16	104	NA		0.81	8	NA	
P08	F/56	Astrocytoma/ III	IDH1*	0.56	41	1.75	16	0.00	999	0.84	26	0.68	9	1.25	16
P09	F/44	TBC	TBC	2.72	11	5.47	18	0.00	999	2.40	29	1.19	6	3.95	22
P10	M/24	TBC	TBC	1.37	16	3.57	12	0.25	68	0.98	32	0.78	8	2.63	11
P11	M/36	TBC	TBC	0.97	47	1.10	36	0.00	999	0.57	54	1.14	11	1.47	19
C01	F/30	-	-	5.75	5	8.71	5	0.24	86	0.83	30	0.34	20	0.14	188
C02	M/35	-	-	4.83	7	7.45	5	0.09	270	0.73	36	0.27	28	0.58	48
C03	F/24	-	-	5.33	6	7.07	6	0.24	92	0.40	67	0.40	17	1.17	32
C04	F/25	-	-	4.50	9	4.47	16	0.73	31	0.00	999	0.61	14	0.91	95
C05	M/24	-	-	4.16	6	5.06	5	0.30	56	0.56	33	0.32	19	0.43	45

Figure 2: Summary table for glutamate (Glu), GABA and lactate (Lac) across glioma patients (n = 11) and healthy volunteers (n = 5). Absolute concentrations reported (mM) as well as Cramér-Rao Lower Bounds (CRLBs) of fitting estimation (%). NA = Not Applicable (Data not acquired), TBC = To Be Confirmed (Awaiting Resection and Histology) and * = Scanned post-operatively

[4] LCModel Fits

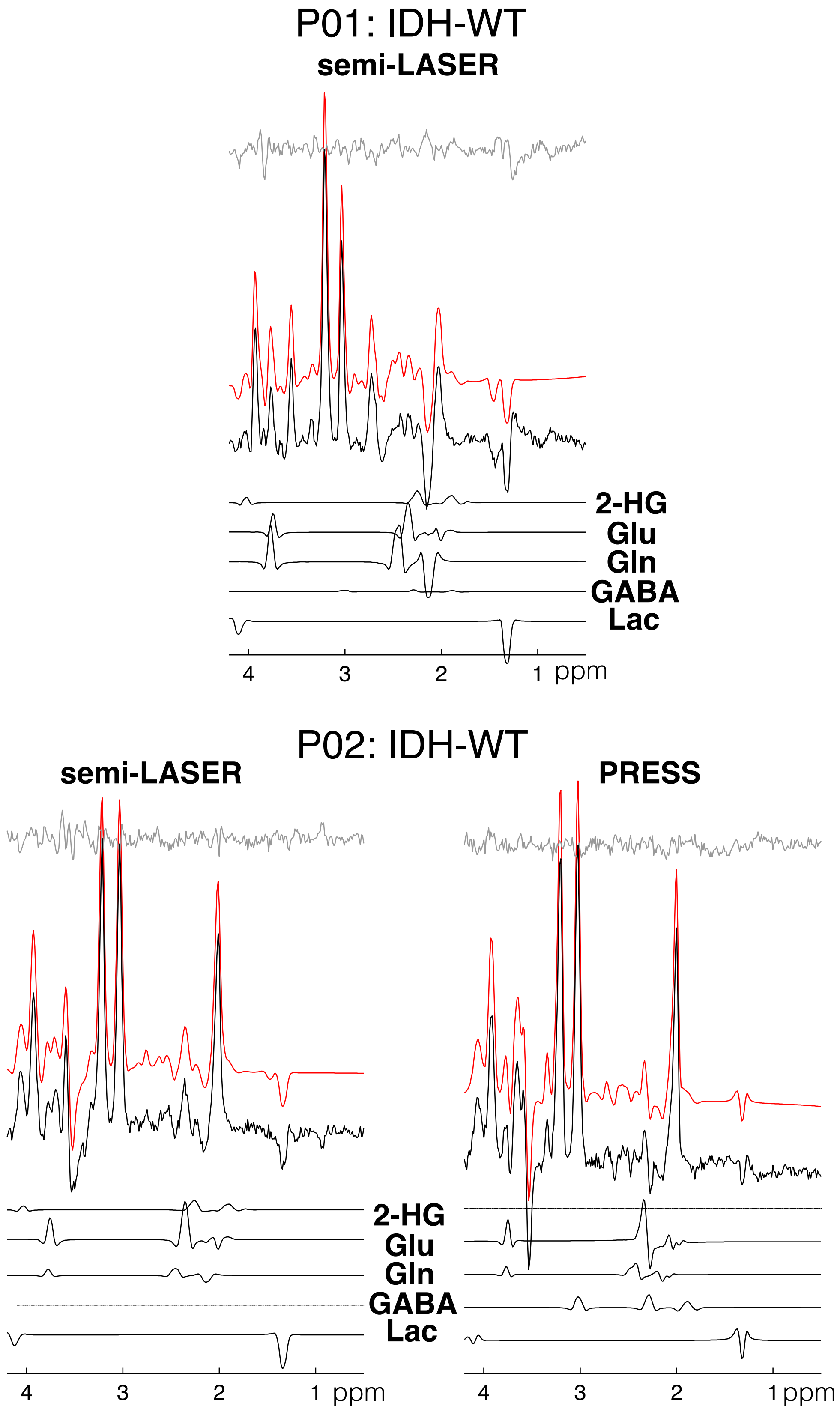

Figure 3: LCModel fitting for the confirmed IDH-WT (wild-type) tumours P01 and P02. Red Line = LCModel fit

P04: IDH1

semi-LASER

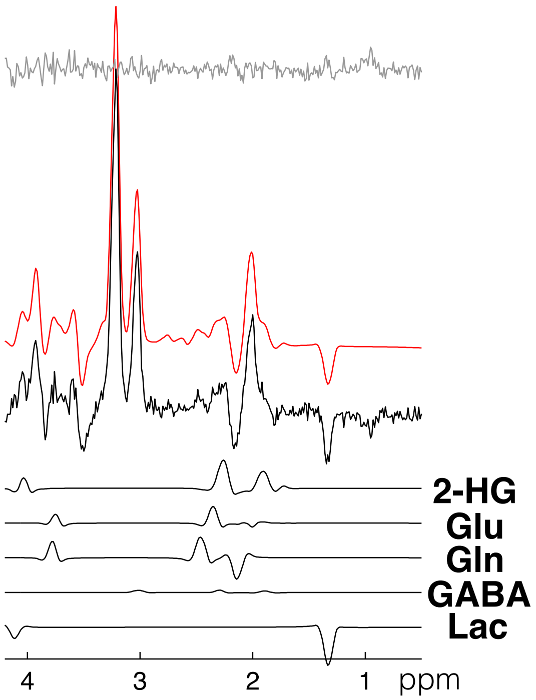

P05: IDH1

semi-LASER

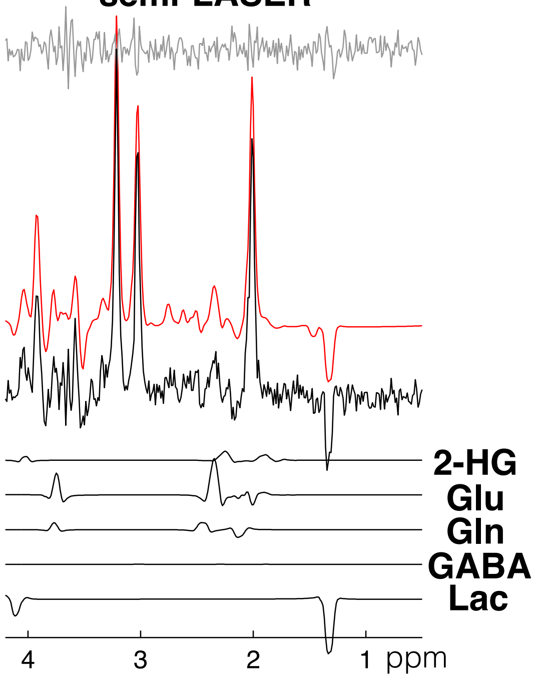

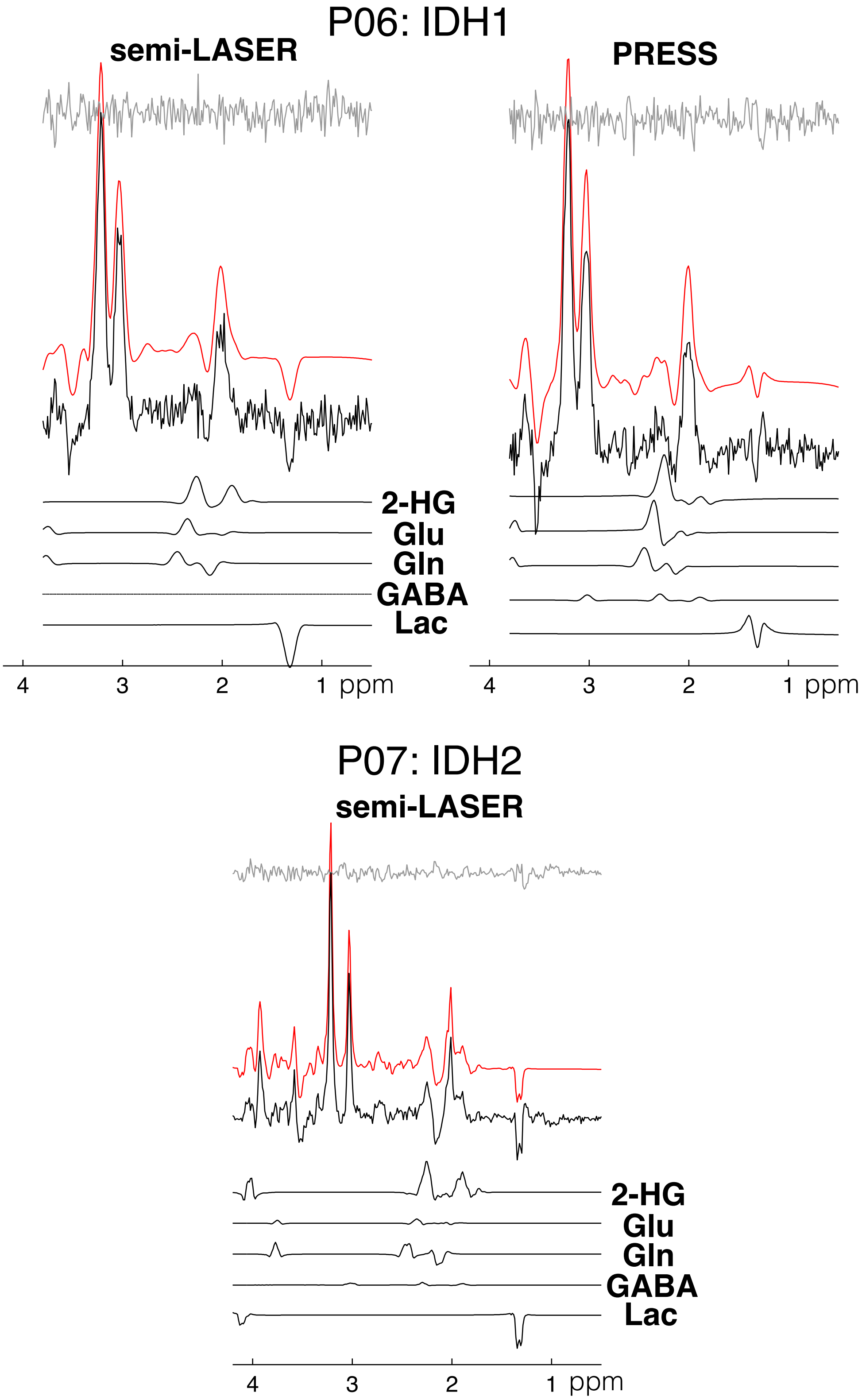

Figure 4: LCModel fitting for the confirmed IDH-mutated tumours P04-7. P07 is confirmed as IDH2 mutation, note the large 2-HG concentration assignment in this patient. Red Line = LCModel fit

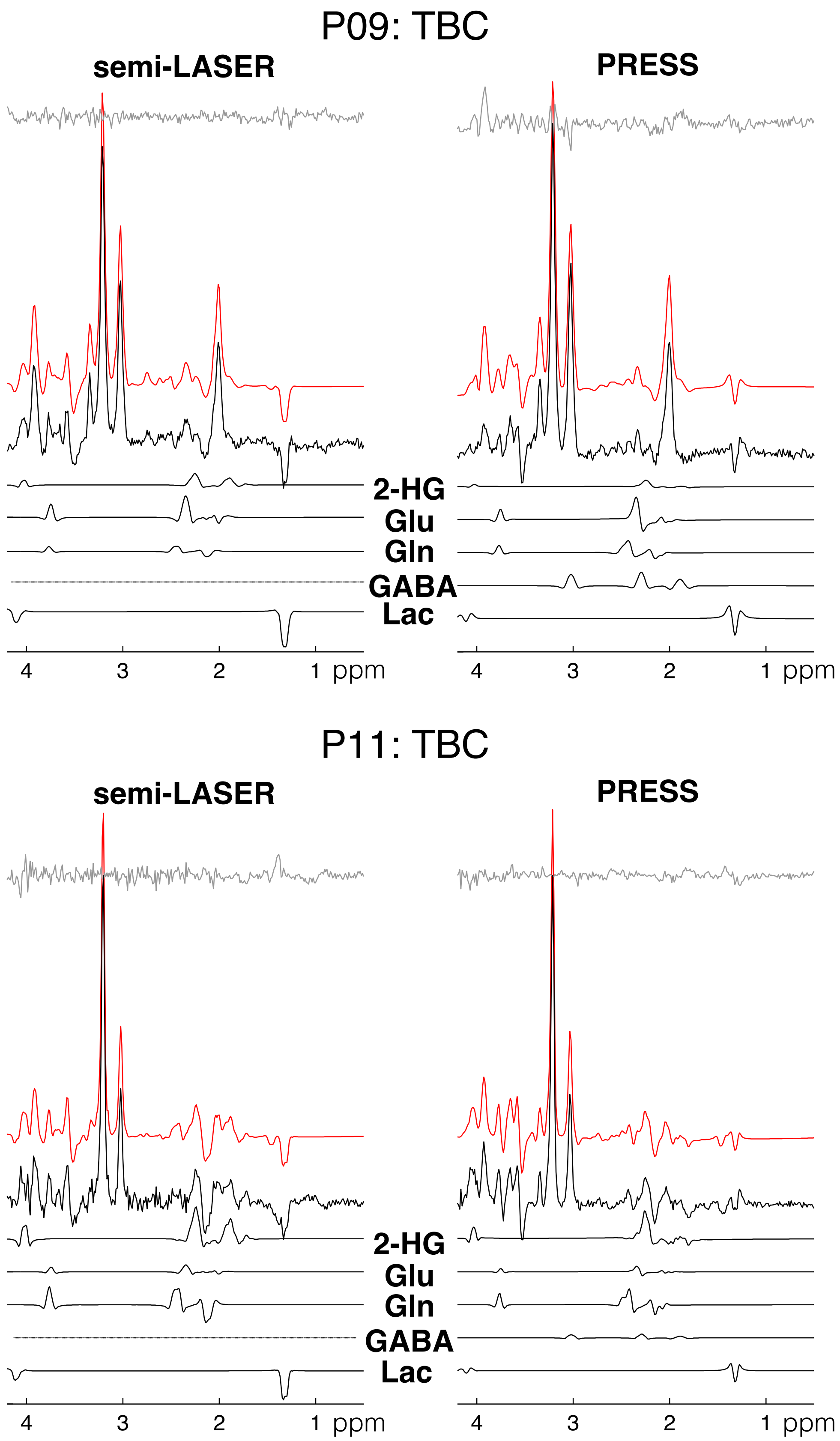

Figure 5: LCModel fitting for the unconfirmed tumours (TBC) P09 and P11. Peaks are seen at 1.9 ppm in the semi-LASER spectra of both patients indicating presence of 2-HG. Red Line = LCModel fit
